# Citation integrity in the AI era: a focused evidence map and layered verification framework for gastroenterology and hepatology publishing

**DOI:** 10.1093/gastro/goag098

**Published:** 2026-09-09

**Authors:** Eun-Ae Jung, Jeong-Ju Yoo, Sang Gyune Kim, Young Seok Kim

**Affiliations:** Medical Library, Soonchunhyang University Bucheon Hospital, Bucheon, 14584, Republic of Korea; Division of Gastroenterology and Hepatology, Department of Internal Medicine, Soonchunhyang University Bucheon Hospital, Soonchunhyang University School of Medicine, Bucheon, 14584, Republic of Korea; Division of Gastroenterology and Hepatology, Department of Internal Medicine, Soonchunhyang University Bucheon Hospital, Soonchunhyang University School of Medicine, Bucheon, 14584, Republic of Korea; Division of Gastroenterology and Hepatology, Department of Internal Medicine, Soonchunhyang University Bucheon Hospital, Soonchunhyang University School of Medicine, Bucheon, 14584, Republic of Korea

Generative artificial intelligence (AI) is increasingly used to draft manuscripts, summarize evidence, and support literature searches. Citation-integrity concerns extend across disciplines but are especially consequential in gastroenterology and hepatology, where evidence syntheses and guidelines shape clinical decisions. Fabricated or contextually unsupported citations may distort risk estimates, eligibility criteria, and clinical recommendations and create false evidentiary links in the scholarly record [1]. Current evidence, however, does not show a higher prevalence of citation errors in these specialties. We therefore conducted a focused evidence map of AI-generated reference errors and related reference-integrity evidence and developed a layered editorial framework distinguishing bibliographic validity from evidentiary support.

A focused, librarian-assisted, title-restricted search of PubMed, Web of Science, and the Research Information Sharing Service (RISS) was conducted to identify reports addressing fabricated, inaccurate, or unreliable references generated by AI tools. The primary search was designed to prioritize specificity and support a focused evidence map rather than a comprehensive systematic review. To capture broader audits of published literature that might not include AI-related terms in their titles, we additionally performed a targeted supplementary PubMed search for fabricated or fake references or citations combined with terms related to audits, published literature, or preprints, without requiring an AI-related term. The full search strategies, eligibility criteria, screening process, and data extraction items are provided in the Supplementary Methods.

Nineteen records published between 2023 and 2026 were included (Supplementary Fig. S1), and their characteristics are summarized in Supplementary Table S1. Twelve contained original quantitative data or database audits, whereas the remainder were editorials, comments, perspectives, or news analyses addressing publication ethics and editorial policy. Most empirical studies evaluated ChatGPT, GPT-3.5, GPT-4, Bard/Gemini, or other AI chatbots in medical or scholarly writing tasks [2, 3]. Verification commonly relied on PubMed, Google Scholar, Web of Science, digital object identifier (DOI) matching, Crossref and other bibliographic checks, or expert review.

Across empirical studies, reference-error rates were substantial but task-dependent (Supplementary Table S2). Fabricated citations accounted for 55% of GPT-3.5 citations and 18% of GPT-4 citations in a multidisciplinary evaluation [4], while hallucination rates in a systematic-review replication task were 39.6%, 28.6%, and 91.4% for GPT-3.5, GPT-4, and Bard, respectively [5]. Among ChatGPT-generated medical references, only 7% were authentic and accurate [6], and another medical question-answering study found that 69% of evaluated references were fabricated [7]. More recent models did not eliminate the problem: in health care simulation manuscripts generated by ChatGPT-4 and ChatGPT o1, 60.4% of references were accurate, but only 22.2% of in-text citations were highly relevant [8]. Complementing these model-output studies, Topaz *et al.* [9] audited 2,471,758 published papers and identified 4,046 fabricated references across 2,810 papers, with the proportion of affected papers increasing from ∼1 in 2,828 in 2023 to 1 in 277 in early 2026. This audit demonstrates a growing real-world integrity problem but does not establish its cause, because paper mills, intentional misconduct, uncritical AI use, and other verification failures may all contribute. It supports scalable reference-existence and metadata checks, which establish bibliographic validity but cannot determine whether a genuine reference contextually supports the claim for which it is cited.

These findings support a three-layer editorial verification framework (Fig. 1). Layers 1 and 2 establish bibliographic validity by confirming reference existence and metadata accuracy, whereas Layer 3 assesses whether the cited article contextually supports the specific in-text claim. A genuine and correctly described article may still be irrelevant, address a different population or outcome, contradict the claim, or support only a weaker conclusion. Contextual verification is particularly important in reviews, guidelines, meta-analyses, bibliometric analyses, and clinical practice updates, in which citations may function as evidence or data; when unsupported citations are used as data, the problem may resemble fabrication [10]. In gastroenterology and hepatology, risk-based review could prioritize citations supporting surveillance intervals, endoscopic interventions, transplant candidacy or timing, and treatment selection in digestive oncology. Automated tools may assist in triaging potential claim–citation mismatches, particularly in evidence-intensive manuscripts, but human editorial judgment remains essential for adjudicating contextual citation support. Table 1 operationalizes each layer by specifying the verification question, tools, automation level, responsible party, editorial stage, and potential action.

**Figure 1:**
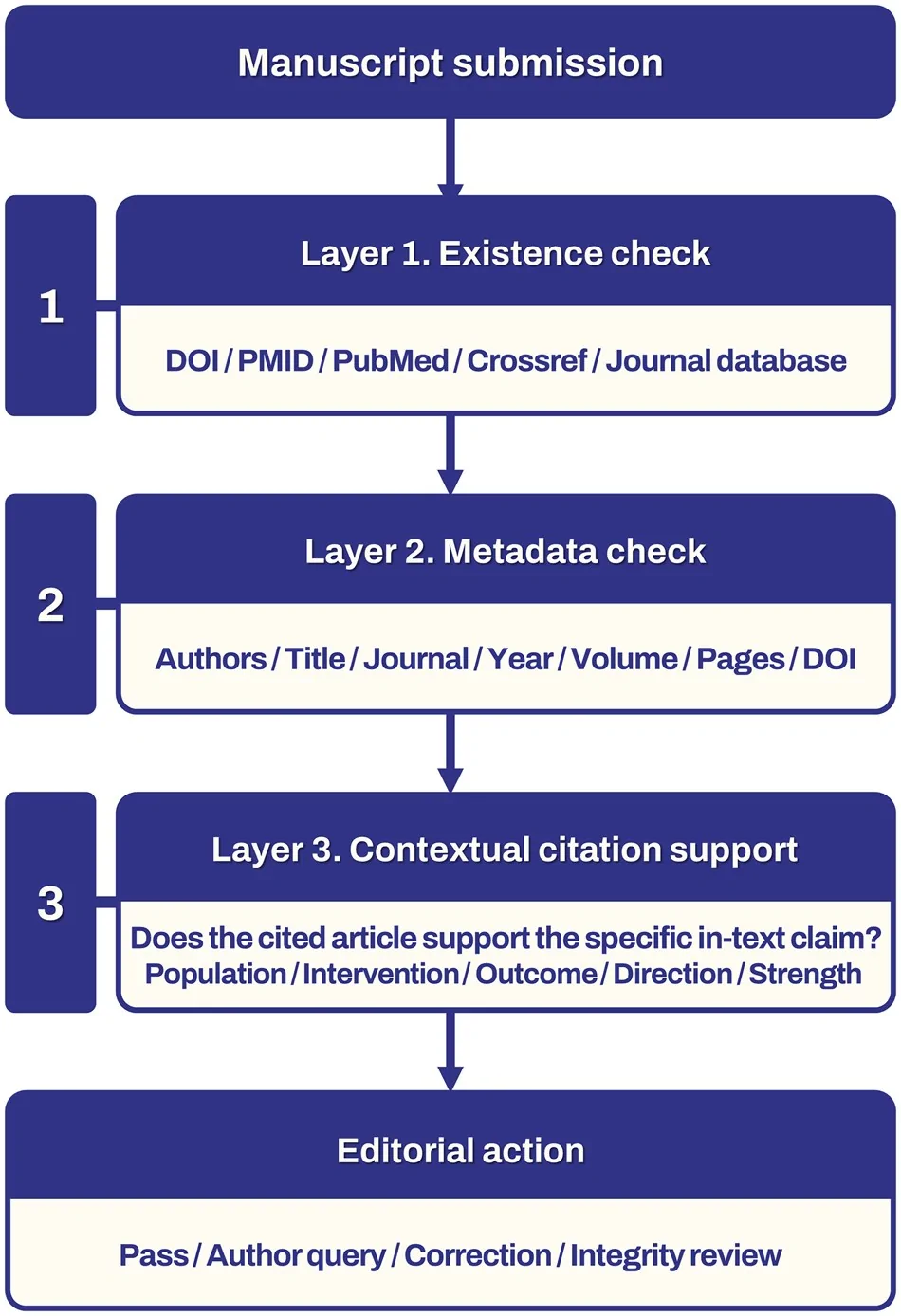
Three-layer pre-publication editorial verification framework for reference integrity. The first two layers establish bibliographic validity by confirming reference existence and metadata accuracy. The third layer assesses evidentiary validity by determining whether the cited article supports the specific in-text claim. A reference may be genuine and bibliographically correct yet be irrelevant, contradictory, applicable to a different population or outcome, or insufficient to support the strength of the authors’ conclusion. Automated tools may assist with screening and triage, but human editorial oversight remains essential for adjudicating contextual citation support.

**Table 1:** Pre-publication operational workflow for layered editorial verification of references^a^

Layer	Verification question	Tools or databases	Automation level	Responsible party	Editorial stage	Possible action
1. Reference existence	Does the cited work exist, and does the DOI, PMID, or other identifier resolve to a genuine publication?	DOI and PMID resolvers; PubMed; Crossref; OpenAlex; publisher or journal databases; supplementary searches for non-indexed sources	High for references with structured identifiers; moderate for references without identifiers	Editorial office, supported by automated screening	At submission, before peer review	Pass; query, correct, or remove; integrity review if systematic
2. Metadata accuracy	Do the authors, title, journal, year, volume, pages, and identifiers match authoritative records?	PubMed and Crossref metadata; publisher APIs or journal websites; bibliographic databases	High for structured records; moderate when metadata are incomplete or inconsistently formatted	Editorial office or copyeditor; author confirmation when needed	At submission or pre-review screening; repeated during copyediting if necessary	Correct metadata; query the author; hold until resolved
3. Contextual citation support	Does the cited article support the specific in-text claim, including the relevant population, intervention or exposure, outcome, direction, magnitude, and strength of inference?	Article abstract and full text; publisher platforms; citation-context retrieval or claim–citation comparison tools; subject-matter review	Low to moderate; automated triage may assist, but human adjudication is required	Handling editor and peer reviewer; subject-matter expert when needed	Editorial assessment and peer review; prioritized for evidence-intensive or high-risk claims	Revise the claim; replace or add evidence; integrity review if material or pervasive

a Editorial actions should be proportionate to the frequency, materiality, and apparent intent of the identified error. Isolated and readily correctable discrepancies should be distinguished from systematic fabrication or repeated contextual misrepresentation. Automated screening can produce both false-positive and false-negative results and may provide incomplete coverage of books, websites, guidelines, grey literature, and non-indexed sources. Failure to identify a reference through automated screening should trigger manual review rather than be treated as proof of fabrication.

Figure 1 and Table 1 describe the pre-publication manuscript workflow, which should be complemented by system-level infrastructure and post-publication pathways. Topaz *et al.* [9] proposed automated reference verification before peer review, incorporation of reference-integrity metadata into indexing services, retrospective screening of published literature, and recognition of fabricated references as a distinct research-integrity category. These measures could institutionalize existence and metadata verification at scale. Retrospective screening and, when warranted, correction, expression of concern, or retraction are complementary post-publication pathways. However, these system-level measures do not replace Layer 3, because confirming that a reference exists and is bibliographically correct does not establish that it supports the corresponding claim.

This focused evidence map was designed to prioritize specificity rather than completeness. Its title-restricted primary search may have missed studies that did not use both AI-related and reference-related terms in their titles, and the targeted supplementary search did not eliminate this possibility. Outcome definitions varied across studies. Web-connected, retrieval-augmented, and agentic systems may reduce reference-existence and metadata errors; however, error rates remain model-, version-, task-, and date-specific and should not be generalized across evolving systems. Most included records were not specialty-specific; therefore, the relevance to gastroenterology and hepatology reflects the field’s reliance on evidence-intensive and clinically consequential publications rather than a demonstrated excess risk of citation errors. Finally, the proposed workflow has not been prospectively evaluated for accuracy, workload, false-positive burden, or effects on editorial decisions. The findings should therefore be interpreted as a structured overview of selected evidence with editorial implications, not as an exhaustive systematic review.

AI may improve scholarly efficiency, but it also exposes a vulnerable point in the publication pipeline. Reference existence and accurate metadata are necessary but insufficient safeguards. In evidence-intensive fields, such as gastroenterology and hepatology, citation integrity also requires confirmation that the cited article supports the specific claim. A layered, human-supervised workflow that distinguishes bibliographic validity from evidentiary validity is needed to protect the reliability of digestive disease scholarship and the clinical recommendations built upon it.

## Supplementary material

Supplementary material is available at *Gastroenterology Report* online.

## Authors’ contributions

J.-J.Y. conceived the study and developed the editorial framework. E.-A.J. and J.-J.Y. designed the methodology, extracted the data, and performed the evidence mapping. E.-A.J. conducted the literature search and data curation. J.-J.Y., S.G.K., and Y.S.K. interpreted the findings. J.-J.Y. drafted the manuscript, and all authors critically revised it. All authors read and approved the final manuscript.

## Supplementary Material

goag098_Supplementary_Data

## Data Availability

All data underlying this focused evidence map are provided in the Supplementary Methods and Supplementary Tables. No additional dataset was generated.
