# Supplementary material for: Citation integrity in the AI era: a focused evidence map and layered verification framework for gastroenterology and hepatology publishing
Source: Gastroenterol Rep (Oxf). 2026 Sep 9;14:goag098. doi: 10.1093/gastro/goag098 (PMC13557618; doi:10.1093/gastro/goag098)
Supplement: Supplementary file 1 [file goag098_supplementary_data.zip › Supplementary material_20260809.docx]

# Supplementary Material

**Supplementary Methods**

**Search strategy, supplementary search, and scope**

A librarian-assisted literature search was performed to identify published reports addressing fabricated, hallucinated, inaccurate, or unreliable references or citations generated by artificial intelligence (AI), generative AI, large language models (LLMs), or chatbot systems, as well as published-literature audits relevant to the detection and editorial management of fabricated references. The following databases were searched: PubMed, Web of Science, and RISS. The search was designed to maximize specificity for reference- or citation-related hallucination by restricting key concepts to the title field.

The search strategy was as follows: (Search Date: 11-June-2026)

("hallucinat*"[Title] OR "fabricat*"[Title] OR "fake"[Title] OR "fraudulent"[Title] OR

"error*"[Title] OR "reliabilit*"[Title] OR "integrit*"[Title])

AND

("Artificial Intelligence"[MeSH Terms] OR "artificial intelligence*"[Title] OR

"AI"[Title] OR "GenAI"[Title] OR "OpenAI"[Title] OR

"large language model*"[Title] OR "LLM"[Title] OR

"ChatGPT"[Title] OR "GPT"[Title])

AND

("quotation*"[Title] OR "reference*"[Title] OR "citation*"[Title])

The final search output provided by the library contained 18 records. Because the objective of this Commentary was to map the available evidence and develop an editorial verification framework rather than to estimate a single pooled effect size, no formal meta-analysis was performed.

To complement the AI-focused primary search, we performed a targeted supplementary PubMed search for audits of fabricated references in published literature. This search did not require an AI-related term and used the same publication-period cutoff as the primary search.

( (fabricat*[Title] OR fake[Title]) AND (reference*[Title] OR citation*[Title]) AND ( audit*[Title/Abstract] OR "published literature"[Title/Abstract] OR preprint*[Title/Abstract] ) ) AND ("2023/01/01"[Date - Publication] : "2026/06/11"[Date - Publication])

The supplementary search retrieved two records. One was the Nature news report by Naddaf, which was already included in the primary evidence map, and the other was the Lancet published-literature audit by Topaz et al., which was newly included.

**Eligibility criteria**

Records were eligible if they addressed at least one of the following:

1. fabricated or non-existent references generated by AI, GenAI, LLMs, or chatbots;
2. inaccurate bibliographic metadata in AI-generated references, including errors in authorship, title, journal, year, volume, page numbers, DOI, PMID, or hyperlinks;
3. contextual inaccuracy or irrelevance of AI-generated in-text citations;
4. ethical, editorial, or research-integrity implications of AI-generated fabricated citations.
5. large-scale audits detecting fabricated references in published scholarly or biomedical literature, irrespective of whether the mechanism responsible for individual fabricated references could be established.

Original empirical studies, database audits, research letters, editorials, comments, perspectives, and news analyses were eligible for qualitative evidence mapping. Quantitative extraction was performed only for studies or reports that provided a denominator for generated references, citations, manuscripts, papers, or other auditable units.

Records were excluded if they discussed general AI hallucination without addressing references or citations; focused only on AI authorship, plagiarism, or text generation without citation-related outcomes; or lacked sufficient information to determine relevance to reference verification.

**Screening and selection**

The primary search yielded 18 de-duplicated records. The targeted supplementary search identified two records, of which one was already included in the primary search and one represented an additional eligible record. After de-duplication, 19 unique records were screened. One author (EAJ) screened all records and assessed eligibility, and a second author (JJY) reviewed the eligibility decisions and any uncertain records. Screening was not performed independently in duplicate. Any disagreements were resolved through discussion and consensus. All 19 records were included in the focused qualitative evidence map; 12 contained original quantitative data, database-audit findings, or extractable denominators and were included in the quantitative evidence table.

**Data extraction**

For each included record, the following variables were extracted when available: first author, year, journal, article type, field or task, evidence-source category, AI model or system evaluated, number of generated or audited references, verification method, definition of fabricated or inaccurate reference, frequency of fabricated references, frequency of partially inaccurate references, frequency of accurate references, citation relevance, whether AI causation was directly established or not assessable, and proposed safeguards.

For original empirical studies, quantitative outcomes were extracted preferentially at the reference or citation level. When multiple AI models or prompt conditions were evaluated, model-specific results were extracted when available. When only summary accuracy scores were reported, the reported score or range was recorded.

**Reference verification**

Following revision, two authors (EAJ, JJY) manually verified all references cited in the main article and all records included in Supplementary Tables 1 and 2. Each record was searched in PubMed and Crossref, and its title, authors, journal, publication year, volume and issue, page range or article number, DOI, and PMID were compared with the journal or publisher website. Discrepancies were resolved by consensus. DOI and PMID identifiers were reported when available; records without a PMID were designated as not indexed in PubMed. CiteWell was not used for this verification.

**Evidence synthesis**

We first distinguished studies that directly evaluated references generated by specific AI models from audits of references already present in published literature. The latter can establish the presence and prevalence of fabricated references in the scholarly record but cannot, by themselves, determine whether individual references originated from AI use, paper mills, intentional misconduct, or other mechanisms. Because the included records varied substantially in article type, field, AI model, prompt design, verification method, and outcome definitions, the findings were synthesized descriptively.

For the evidence synthesis, we distinguished bibliographic validity from evidentiary validity. Bibliographic validity comprised reference existence and accurate bibliographic metadata. Evidentiary validity, termed contextual citation support in our framework, referred to whether the content of the cited article supported the specific in-text claim, considering the relevant population, intervention or exposure, outcome, direction of association, and strength of inference. Contextual mismatch included irrelevant or contradictory citations, mismatches in population or outcome, and claims that were broader or stronger than the cited source supported.

Accordingly, reference-related errors were categorized into three levels:

1. non-existent or fabricated references;
2. existing references with inaccurate bibliographic metadata; and
3. existing and bibliographically accurate references that did not contextually support the corresponding in-text claim.

When included studies reported citation relevance or contextual support, we extracted the authors’ reported assessments; we did not independently re-adjudicate every claim–citation pair across the included studies. This three-level taxonomy formed the basis of the editorial verification framework shown in Figure 1 of the main article.

**Operationalization of the editorial framework**

The three-level taxonomy was translated into a proposed editorial workflow by specifying the verification question, applicable tools and databases, feasible level of automation, responsible party, editorial stage, and potential action at each layer. Automation was considered most feasible for identifier-based existence and metadata checks. For contextual citation support, automated approaches were considered suitable primarily for screening or prioritization, with final adjudication requiring human editorial or subject-matter judgment. The proposed workflow is intended as a pragmatic, human-supervised framework and has not yet been prospectively validated for accuracy, workload, false-positive burden, or effects on editorial decisions.

**Supplementary Figure 1. Flow diagram**

Flow diagram of study selection. The primary search identified 18 records, and the targeted supplementary PubMed search identified two records, one of which duplicated a record from the primary search. After de-duplication, 19 records were included in the qualitative evidence map, of which 12 provided quantitative data, database-audit findings, or extractable denominators.

**Supplementary Table 1. Summary of 19 included records**

| **No.** | **First author** | **Year** | **Journal** | **Article type** | **PMID** | **DOI** | **Field or focus** | **Model/system or evidence source** | **Role in evidence map** |
| --- | --- | --- | --- | --- | --- | --- | --- | --- | --- |
| 1 | Singh Chawla | 2026 | Nature | News | 42156877 | 10.1038/d41586-026-01595-5 | arXiv policy and hallucinated references | AI-generated references | Described policy response to hallucinated references and potential sanctions |
| 2 | Naddaf | 2026 | Nature | News/audit report | 42104128 | 10.1038/d41586-026-00748-w | Biomedical literature audit | Published biomedical papers | Secondary news coverage of the Topaz et al. audit; not treated as an independent quantitative dataset |
| 3 | Bauchner | 2026 | Lancet | Comment | 42107358 | 10.1016/S0140-6736(26)00798-1 | Editorial integrity | AI/fabricated references | Framed fabricated references as an emerging threat to editorial integrity |
| 4 | Resnik | 2026 | Accountability in Research | Ethics analysis | 41833014 | 10.1080/08989621.2026.2645390 | Research misconduct | Generative AI | Argued that hallucinated citations may constitute misconduct when citations function as data |
| 5 | Pastucha | 2026 | Medical Science Monitor | Original study | 41546380 | 10.12659/MSM.950916 | Otorhinolaryngology guideline topics | ChatGPT and Gemini versions | Evaluated reference accuracy across 6 chatbot versions and repeated prompts |
| 6 | Samartzis | 2026 | European Spine Journal | Editorial | 42213181 | 10.1007/s00586-026-10052-w | Spine evidence and clinical decision-making | LLMs | Discussed clinical consequences of false references |
| 7 | Gibney | 2025 | Nature | News | 40921873 | 10.1038/d41586-025-02853-8 | AI hallucination and newer models | GPT-5 and other AI models | Discussed whether newer AI models reduce fabricated citations |
| 8 | Cheng | 2025 | Simulation in Healthcare | Original study | 40772773 | 10.1097/SIH.0000000000000877 | Health care simulation manuscripts | ChatGPT-4 and ChatGPT o1 | Evaluated both reference accuracy and in-text citation relevance |
| 9 | Aljamaan | 2024 | JMIR Medical Informatics | Original study | 39083799 | 10.2196/54345 | Medical AI chatbot prompts | Multiple AI chatbots | Developed Reference Hallucination Score for chatbot-generated references |
| 10 | Chelli | 2024 | Journal of Medical Internet Research | Original study | 38776130 | 10.2196/53164 | Systematic review replication | GPT-3.5, GPT-4, Bard | Compared LLM-generated references with human-conducted systematic reviews |
| 11 | Mugaanyi | 2024 | Journal of Medical Internet Research | Original study | 38578685 | 10.2196/52935 | Cross-disciplinary scholarly writing | ChatGPT-3.5 | Compared citation and DOI accuracy in natural sciences and humanities |
| 12 | Sebo | 2024 | Internal and Emergency Medicine | Research letter | 37979017 | 10.1007/s11739-023-03484-5 | Internal medicine references | ChatGPT-3.5 | Evaluated correctness of 100 ChatGPT-generated references |
| 13 | Walters | 2023 | Scientific Reports | Original study | 37679503 | 10.1038/s41598-023-41032-5 | Multidisciplinary literature reviews | GPT-3.5 and GPT-4 | Quantified fabricated and erroneous bibliographic citations |
| 14 | McGowan | 2023 | Psychiatry Research | Original study | 37499282 | 10.1016/j.psychres.2023.115334 | Psychiatry literature search | ChatGPT and Bard | Demonstrated spontaneous citation fabrication during literature search |
| 15 | Bhattacharyya | 2023 | Cureus | Original study | 37337480 | 10.7759/cureus.39238 | ChatGPT-generated medical content | ChatGPT-3.5 | Quantified fabricated, inaccurate, and accurate medical references |
| 16 | Gravel | 2023 | Mayo Clinic Proceedings: Digital Health | Original study | 40206627 | 10.1016/j.mcpdig.2023.05.004 | Medical question answering | ChatGPT | Evaluated answer quality and fabricated references in medical questions |
| 17 | Picazo-Sanchez | 2026 | Data | Original/database study | Not available | 10.3390/data11050122 | LLM-generated citations and published literature | 9 LLMs and published papers | Generated large BibTeX dataset and assessed fabricated references in published papers |
| 18 | Boretti | 2026 | Energy Research & Social Science | Perspective | Not available | 10.1016/j.erss.2026.104720 | Scholarly integrity and publisher-led verification | Generative AI | Proposed publisher-led verification and human-supervised safeguards |
| 19 | Topaz | 2026 | Lancet | Correspondence / published-literature audit | 42107362 | 10.1016/S0140-6736(26)00603-3 | Reference integrity in published biomedical literature | No generative model evaluated as the source of citations; automated verification system used | Large-scale audit demonstrating the presence and increasing frequency of fabricated references in published biomedical literature; causation was not attributed exclusively to AI |

**Supplementary Table 2. Empirical studies and denominator-based outcomes**

| **First author** | **Year** | **Field/task** | **AI model/system** | **Denominator** | **Verification method** | **Main quantitative outcome** |
| --- | --- | --- | --- | --- | --- | --- |
| Pastucha | 2026 | Otorhinolaryngology guideline topics | 3 ChatGPT versions and 3 Gemini versions | 1,947 references | PubMed, Web of Science, Google Scholar | ChatGPT-4.1 with web search achieved the best accuracy score of 51%; Gemini 2.5 Pro was second at 41%. Totally false references occurred in 1%–22% of ChatGPT references and 0%–26% of Gemini references, depending on model/version. |
| Cheng | 2025 | Health care simulation debriefing manuscripts | ChatGPT-4 and ChatGPT o1 | 303 references; 451 citations | Independent blinded reviewer assessment | 183/303 references were accurate (60.4%). Completely fabricated references accounted for 23.4%, and partially fabricated references for 16.9%. Only 22.2% of in-text citations were highly relevant. |
| Aljamaan | 2024 | Medical chatbot prompts | ChatGPT, Bing, Perplexity, Elicit, SciSpace, Bard | 500 generated references evaluated for hallucination items | PubMed and Google Scholar comparison | ChatGPT 3.5 and Bing had the highest Reference Hallucination Score; Elicit and SciSpace had the lowest. Hallucination was most frequent for reference relevance to prompt keywords, 308/500 (61.6%). |
| Chelli | 2024 | Systematic review replication | GPT-3.5, GPT-4, Bard | 471 references | Comparison with references from human-conducted systematic reviews | Hallucination rates were 55/139 (39.6%) for GPT-3.5, 34/119 (28.6%) for GPT-4, and 95/104 (91.4%) for Bard. Precision was 9.4%, 13.4%, and 0%, respectively. |
| Mugaanyi | 2024 | Scholarly writing in natural sciences and humanities | ChatGPT-3.5 | 102 citations | Verification of citation existence, DOI existence, DOI accuracy, and relevance | 76/102 citations (74.5%) were found to be real. DOI existence was higher in natural sciences than humanities, 39/55 (70.9%) vs 18/47 (38.3%). DOI hallucination was more frequent in humanities than natural sciences. |
| Sebo | 2024 | Internal medicine references | ChatGPT-3.5 | 100 references | PubMed, Google, Google Scholar | 34/100 references were correct, 40/100 were partially correct, and 26/100 were incorrect or probably fake. |
| Walters | 2023 | Multidisciplinary literature reviews | GPT-3.5 and GPT-4 | 636 citations | Multiple databases and websites | 55% of GPT-3.5 citations and 18% of GPT-4 citations were fabricated. Among real citations, substantive bibliographic errors occurred in 43% of GPT-3.5 citations and 24% of GPT-4 citations. |
| McGowan | 2023 | Psychiatry literature search | ChatGPT and Bard | 35 ChatGPT-generated citations in initial trial | PubMed, Google Scholar, hyperlink verification | Only 2/35 citations matched actual manuscripts. Twelve citations resembled real manuscripts but contained errors, and 21 had no clear origin or represented fabricated/pastiche references. |
| Bhattacharyya | 2023 | ChatGPT-generated medical content | ChatGPT-3.5 | 115 references | Medline, Google Scholar, Directory of Open Access Journals | 54/115 references (47%) were fabricated, 53/115 (46%) were authentic but inaccurate, and 8/115 (7%) were authentic and accurate. In the gastroenterology prompt subset, 8/13 references (62%) were fabricated and none were fully accurate. |
| Gravel | 2023 | Medical question answering | ChatGPT | 59 references | Manual search and expert evaluation | 41/59 references (69%) were fabricated despite appearing plausible. |
| Picazo-Sanchez | 2026 | LLM-generated citations and published literature | 9 LLMs and published papers | 74,196 generated BibTeX references; 127,063 references from 3,541 published papers | Automated extraction and manual verification | Large-scale analysis of LLM-generated references and published-paper references identified fabricated bibliographic data in the published literature, including 8 manually verified fabricated references. |
| Topaz | 2026 | Published biomedical literature in the PubMed Central Open Access corpus | Not applicable (published-literature audit) | 2,471,758 papers; 125,615,773 structured references, of which 97.1 million were verified | Automated bibliographic and identifier matching using PubMed, Crossref, OpenAlex, and Google Scholar, with manual validation | A total of 4,046 fabricated references were identified across 2,810 papers. Papers containing at least one fabricated reference increased from approximately 1 in 2,828 in 2023 to 1 in 458 in 2025 and 1 in 277 during the first 7 weeks of 2026. The audit did not establish the cause of individual fabricated references. |
